# HDM2 antagonist MI-219 (spiro-oxindole), but not Nutlin-3 (*cis*-imidazoline), regulates p53 through enhanced HDM2 autoubiquitination and degradation in human malignant B-cell lymphomas

**DOI:** 10.1186/1756-8722-5-57

**Published:** 2012-09-18

**Authors:** Angela M Sosin, Angelika M Burger, Aisha Siddiqi, Judith Abrams, Ramzi M Mohammad, Ayad M Al-Katib

**Affiliations:** 1 Department of Oncology, Barbara Ann Karmanos Cancer Institute (KCI), Detroit, MI, 48201, USA; 2 Department of Pharmacology, Wayne State University School of Medicine (WSU-SOM), Detroit, MI, 48201, USA; 3 Biostatistics Core Facility (KCI), Detroit, USA; 4 Department of Internal Medicine, Hematology-Oncology, WSU-SOM and the Lymphoma Clinic, Van Elslander Cancer Center, St John Hospital and Medical Center, Grosse Pointe Woods, MI, 48236, USA; 5 Division of Hematology and Oncology Department of Internal Medicine, Wayne State University School of Medicine, 540 E. Canfield, 8229 Scott Hall, Detroit, MI, 48201, USA

**Keywords:** HDM2, p53, Apoptosis, MI-219, Nutlin-3, B-cell lymphoma, Small-molecule inhibitor, Autoubiquitination

## Abstract

**Background:**

Lymphomas frequently retain wild-type (wt) p53 function but overexpress HDM2, thereby compromising p53 activity. Therefore, lymphoma is a suitable model for studying the therapeutic value of disrupting the HDM2-p53 interaction by small-molecule inhibitors (SMIs). HDM2 have been developed and are under various stages of preclinical and clinical investigation. Previously, we examined the anti-lymphoma activity of MI-319, the laboratory grade of a new class of HDM2 SMI, the spiro-oxindole, in follicular lymphoma. Since then, MI-219, the clinical grade has become readily available. This study further examines the preclinical effects and mechanisms of MI-219 in a panel of human lymphoma cell lines as well as a cohort of patient-derived B-lymphcytes for its potential clinical use.

**Results:**

Preclinical assessment of MI-219 was evaluated by means of an in vitro and ex vivo approach and compared to Nutlin-3, the gold standard. Characterization of p53 activity and stability were assessed by quantitative PCR, Western blot, and immunoprecipitation. Biological outcome was measured using Trypan blue exclusion assay, Annexin V/PI, PARP and caspase-3 cleavage. Surprisingly, the overall biological effects of Nutlin-3 were more delayed (48 h) while MI-219 triggered an earlier response (12-24 h), predominantly in the form of apoptotic cell death. Using a cell free autoubiquitination assay, neither agent interfered with HDM2 E3 ligase function. MI-219 was more effective in upregulating wt-p53 stabilization compared to Nutlin-3. MI-219, but not Nutlin-3, enhanced the autoubiquitination and degradation of HDM2.

**Conclusions:**

Our data reveals unexpected differences between MI-219 and the well-studied Nutlin-3 in lymphoma cell lines and patient samples. We suggest a novel mechanism for MI-219 that alters the functional activity of HDM2 through enhanced autoubiquitination and degradation. Additionally, this mechanism appears to correspond to biological outcome. Our results provide evidence that different classes of HDM2 SMIs elicit molecular events that extend beyond HDM2-p53 dissociation which may be of biological and potentially therapeutic importance.

## Background

The estimated number of newly diagnosed non-Hodgkin’s lymphoma cases for 2012 is expected to be 70,130 of which 18,940 patients (27%) will fail to survive. This represents a 9.89% increase in diagnosed cases compared to that estimated in 2007. While the expected mortality has marginally decreased from 29.5% in 2007 to 27% in 2012, there is reason for optimism but no reason to celebrate until a complete cure is found [1,2]. Unlike solid tumors which could be treated in situ or removed if diagnosed early, lymphoma cells circulate alongside with normal lymphocytes making treatment options limited. However, the inherent defects within the cells that initiate transformation of normal cells into cancer are similar, as are the mechanisms evading cell death and development of resistance.

One of the most recognized defects is altered functionality of p53, the protein product of the TP53 gene [3]. p53, often considered guardian of genome, is a master transcriptional regulator at the center of complex molecular networks controlling cell proliferation and death. Therefore, the integrity of functional wild-type p53 (wt-p53) tumor suppressor activity is a vital component of normal cellular homeostasis and protection from cancer development. p53 is activated in response to multiple stress signals and activated p53 induces genes involved in biological outcomes such as apoptosis, cell cycle arrest, DNA repair, senescence, and more recently, metabolism [4-6]. Deregulation of p53 by inactivating mutations occurs in approximately 50% of all human cancers, impairing DNA binding and transcription of tumor suppressive target genes [7]. Tumors that possess a mutant p53 (mt-p53) have been shown to evade apoptosis, resist many therapeutic interventions, and exhibit qualities associated with poor prognosis [8-11]. Therefore, maintaining functional wt-p53 activity is central to favorable treatment outcomes in cancer.

Numerous posttranslational mechanisms exist to control p53 activity and ultimately determine its cellular fate. HDM2 (Human Double Minute 2) is a RING finger E3-ubiquitin ligase that acts as the predominant negative regulator of p53. HDM2 is also a transcriptional target gene of p53, creating an autoregulatory feedback loop that regulates p53 activity and stability. HDM2 binds to p53, inactivates its transcriptional activity and facilitates ubiquitin-dependent degradation of p53 by exporting it out of the nucleus [12-14]. Furthermore, p53-independent functions of HDM2 exist and may be influenced through interaction with additional proteins [15]. In addition to its role in regulating functional p53 activity, HDM2 is also capable of self-regulation via autoubiquitination and is thought to degrade itself by means of the proteasome [16,17]. Although this self-regulatory function of HDM2 is well-documented, the control and activation of this function still remains highly elusive. Importantly, HDM2 destabilization is required for proper p53 response. In fact, an early step in the accumulation of p53 in response to stress, DNA damage in particular, is an associated increase in HDM2 autoubiquitination and degradation [18,19].

Overexpression of HDM2 has been shown to facilitate cancer development and progression in several tumor types and is often found in hematological malignancies. It is observevd more frequently in low grade non-Hodgkin’s lymphomas (NHL) (56.5%) than in aggressive NHL (10.8%), with HDM2 elevated even further in patients with advanced clinical stage [20]. Abnormal expression of HDM2 has also been detected in Hodgkin lymphomas (HL) [21]. However, little is known about the prognostic value of HDM2 in lymphomas. Several non-genotoxic small-molecule inhibitors (SMIs) have been developed to block HDM2-p53 with attempts to restore tumor suppressive activity to tumor cells with wt-p53. This is an attractive therapeutic strategy [22], particularly in lymphomas, where p53 mutations account for less than 15% of all cases [23,24], yet wt-p53 remains dysfunctional due to overexpressed HDM2.

Although preclinical assessment of several HDM2 SMIs have demonstrated great promise, it is becoming increasingly clear that multiple molecular mechanisms modulate their anti-cancer efficacy in conjunction with genetic components of the patient’s tumor. More importantly, novel HDM2 SMIs are currently under clinical evaluation in phase I studies, demonstrating the significant impact and clinical relevance of these agents [25]. We previously reported the anti-lymphoma effects of a laboratory grade spiro-oxindole MI-319, in our established WSU-FSCCL cell line and xenograft model [26]. Since then, the clinical grade MI-219 has become readily available. In this study, we assessed the preclinical potential of MI-219 and re-examined selected molecular consequences associated with HDM2 inhibition in lymphoma cell lines and a cohort of malignant patient-derived B-lymphocytes. Our data uncovers unexpected differences between MI-219 compared to archetypical Nutlin-3. The results suggest a crucial role of mediating enhanced HDM2 activity towards itself, in conjunction with wt-p53 reactivation, which affects MI-219 sensitivity in this tumor type. More in-depth understanding of how HDM2 SMIs impact the myriad of biological processes conducted by p53 in lymphoma cells is necessary in order to in maximize their therapeutic exploitation.

## Results

### Characteristics of patient sample

To extend our findings previously investigated [26], we determined the efficacy of MI-219 and Nutlin-3 in a number of fresh lymphoma patient samples ex vivo. Over the course of 3 years (2009–2011), analyzable tumor samples obtained from peripheral blood of 11 patients with B-cell lymphoma in leukemic phase were enriched and exposed to increasing concentrations of MI-219 or Nutlin-3 for up to 72 h. Priority of assays performed was dependent upon the total number of available isolated and purified cells and was carried out in the following order: chromosomal karyotyping > determination of p53 status(wild-type [wt] or mutant [mt]) > cell viability post-exposure to Nutlin-3 or MI-219 > Western blot detection of selected p53 target proteins of treated cells. Patient characteristics are shown in Table 1. Eight patients had been clinically diagnosed with small lymphocytic lymphoma (SLL)/chronic lymphocytic leukemia (CLL) and three with marginal zone lymphoma (MZL). Male: female ratio was 5: 6; and median age was 69 years (range 62–86). Of the eight SLL/CLL patients, six exhibited 13q- and the remaining two expressed trisomy 12. All patients, except one (patient #9) exhibited wt-p53. Patient # 9, with 17p- chromosomal abnormality detected in 9% of cells contained a p53 mutation (Lys132Arg). The group of 11 patients represents indolent non-Hodgkin’s lymphoma associated with a lengthy disease course of slowly proliferating lymphoma cells which eventually leads to enlargement of lymph nodes (lymphadenopathy) and bone marrow failure (manifested as anemia, thrombocytopenia and/or neutropenia) secondary to bone marrow replacement by the lymphoma cells thereby inhibiting bone marrow function. No patient was being actively treated at the time of this study; although several patients had been previously treated. 

**Table 1:** Characteristics of lymphoma patient samples

**Patient**#	**AGE**	**SEX**	**RACE**	**TYPE OF LYMPHOMA**	**CYTOGENETICS/KARYOTYPE**	**p53 status Exons 5-9 (cDNA)**
1	62	M	Caucasian	MZL	Normal/46,XY [23]	wt
2	76	M	Caucasian	SLL/CLL	84.5%-trisomy 12	wt
					15.5%- deletion of 17p	
					11%-loss of lgH locus	
3	86	M	Caucasian	MZL	Normal/46, XY [23]	wt
4	71	F	Caucasian	SLL/CLL	67.5%-trisomy 12	wt
5	64	F	African-American	SLL/CLL	86.5%-deleted 13q	wt
6	72	M	Caucasian	MZL	45%-t(2;7)(p12;q21-22)	wt
7	64	F	Caucasian	SLL/CLL	54.5%- deleted 13q	wt
8	81	F	Caucasian	SLL/CLL	58.5%- deleted 13q	wt
9	69	F	Caucasian	SLL/CLL	51%- deleted 13q	mt
					9%- deleted 17p	(Lys132Arg)
10	62	M	Caucasian	SLL/CLL	23.5%- deleted 13q	wt
					26%-31gH copies	
11	69	F	Caucasian	SLL/CLL	72%- deleted 13q	wt

### Effect of HDM2 inhibition in patient-derived B lymphoma cells

**Figure 1:**
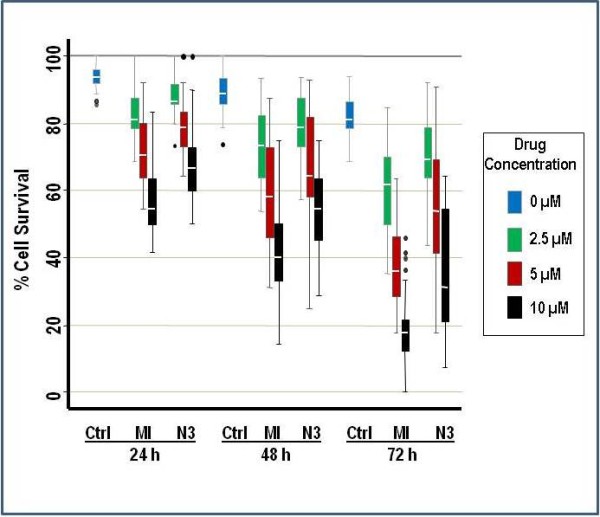
**Reduction of cell survival in patient-derived lymphoma cells exposed to HDM2 SMIs.** Box plots show percent survival compared to control for isolated primary lymphoma cells following exposure to increasing concentrations of MI-219 and Nutlin-3 at indicated time points. Survival is expressed as a percentage of live cells detected by Trypan blue exclusion relative to the total number of cells from each of the 11 patients ranging from 2 to 6 replicates per patient. The horizontal lines within the boxes represent the median while the upper and lower lines of the box endorse the 25th and 75th interquartile range (IQR). The upper- and lower-most lines extend to cover points within 1.5 times the IQR and circles outside of the lines indicate outliers. A mixed effects analysis of variance was used where the drug, concentration and time were defined as fixed effects; patient and replication were defined as random effects. Holm’s procedure was used to adjust for multiple comparisons. Significant overall differences between MI-219 and Nutlin-3 treatments were observed (p < 0.001).

Enriched primary B-lymphocytes were analyzed for cell viability. Both MI-219 and Nutlin-3 induced concentration- and time-dependent decreases in cell viability in isolated and purified primary lymphoma cells. A comprehensive biostatistical analysis was performed on n = 11 lymphoma patient samples to determine whether there were significant differences between Nutlin-3 and MI-219 and the extent of their effects on cell viability. The mean cell survival for each combination of drug, concentration, and time is shown in Figure 1 along with standard errors. The results demonstrate that overall, MI-219 is significantly more effective (p < 0.001) at reducing the cell viability of primary lymphoma cells than Nutlin-3.

**Figure 2:**
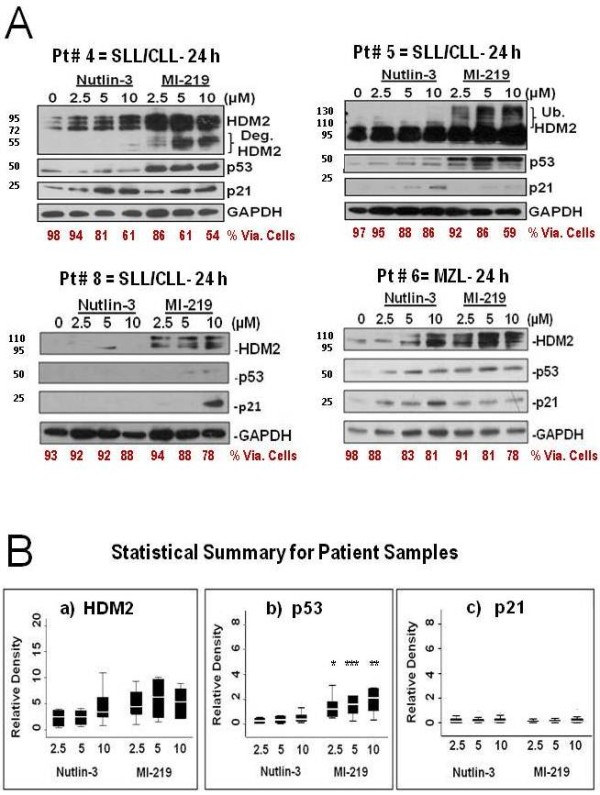
**Upregulation of p53 protein predicts efficacy and biological response to MI-219 in primary lymphoma cells. A**) Western blots of the upregulation of p53 and its target proteins upon HDM2 inhibition after 24 h in primary lymphoma cells isolated from four patients. **B**) Box plots show the cumulative biostatistical analyses of p53 target protein expression levels to estimate response predictors to HDM2 SMIs in primary B-lymphocytes isolated from lymphoma patients (n = 10). Western blots for each patient (n = 10) for each drug, dose, and time point for each protein detected. Quantification of Western blot bands (relative density) was calculated using ImageJ and normalized to internal control (GAPDH). Fold increase or decrease was calculated by standardizing each treatment as a ratio to the control. MI-219 is statistically more effective than Nutlin-3 (p = 0.001) overall regardless of p53 target protein or time point when main effects for drug, concentration, protein and time were fitted without interactions. Upregulation of p53 was statistically greater upon exposure to MI-219 than for Nutlin-3 at 24 h for equivalent concentration; 2.5 μM [*p = 0.05]; 5.0 μM [***p = 0.02] and 10 μM [**p = 0.03] shown in Figure 2-Bb).

HDM2 inhibitors activate the p53 pathway in primary lymphoma cells. Both Nutlin-3 and MI-219 induced variable HDM2, p53 and p21 protein expression in purified patient-derived B-lymphocytes (Figure 2)*.* Of special interest is the observation that MI-219, but not Nutlin-3, induced both higher and lower molecular weight species of HDM2. These molecular changes were best captured at 24 h and Western blots for 3 patients with SLL/CLL and 1 with MZL lymphoma are shown in Figure 2A. A statistical analysis summary for changes in the induction of p53-target proteins following exposure to HDM2 SMIs in patient samples is shown in Figure 2B*.* Cumulatively, MI-219 was more effective than Nutlin-3 (p = 0.001) in the upregulation of p53, p21, and HDM2 protein levels in primary B-lymphoma cells. At 24 h, expression of p53 protein was significantly induced with MI-219 compared to Nutlin-3 at all concentrations and was the largest contributor to the overall significant difference between the two treatments (Figure 2B)*.*

### Induction of apoptotic cell death in wt-p53 lymphoma cell lines

**Figure 3:**
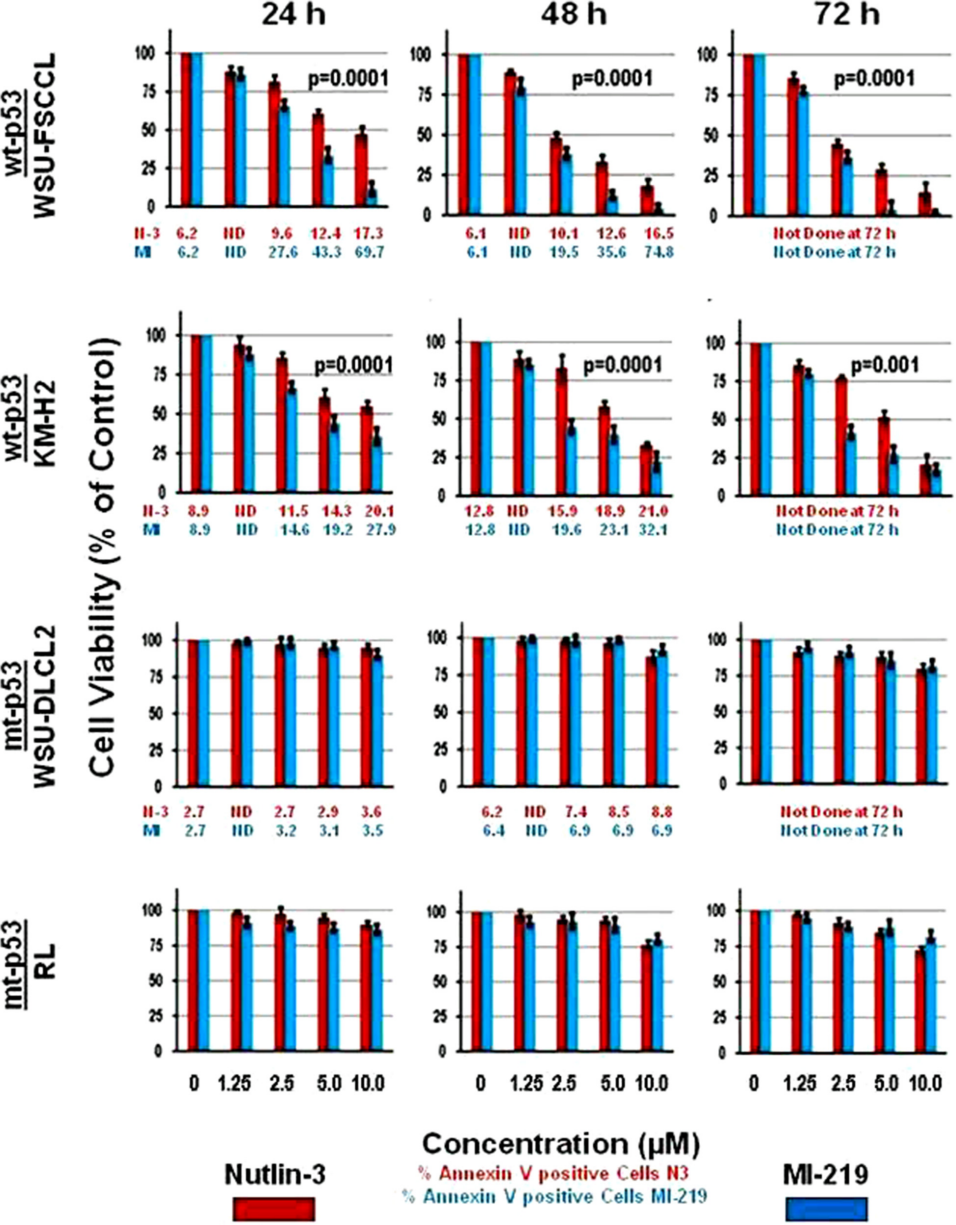
**Biological response of lymphoma cell lines to HDM2 SMIs.** Nutlin-3 (Red bars) and MI-219 (Blue bars) represent cell viability for 4 cell lines exposed to increasing concentrations of HDM2 SMIs for up to 72 h. Comparison for overall differences across equivalent concentrations were significant between the two HDM2 SMIs (ANOVA; p = 0.001) for each time period (24 to 72 h) in both wt-p53 cell lines. Numerical values located under the graphs represent percentage of Annexin V positive cells for Nutlin-3 (red print) and MI-219 (blue print). MI-219- induced increases in Annexin V positive WSU-FSCCL cells at 24 and 48 h was significantly greater than that for Nutlin-3 (p = 0.0001) within the same cell line. For KM-H2 cells, the difference was significant only at 24 h (p = 0.026). Columns and error bars represent Mean ± S.D. of three independent experiments.

A series of experiments were performed using both HDM2 SMIs in two wt-p53 and two mt-p53 lymphoma cell lines. As expected, cell death of mt-p53 cell lines RL and WSU-DLCL_2_ were not significantly affected by exposure to either HDM2 SMI at concentration up to 10 μM (Figure 3). In wt-p53 WSU-FSCCL and KM-H2 cells, the overall effect of MI-219-induced cell death was significantly greater than that of Nutlin-3. This was seen as early as 24 h following initiation of treatment (p = 0.0001). The apoptotic effect was more evident in the non-Hodgkin’s lymphoma WSU-FSCCL cell line than in the Hodgkin lymphoma KM-H2 cell line. MI-219 treatment in WSU-FSCCL cells led to complete elimination of cells at the end of 72 h at 5 μM and 10 μM concentrations unlike that seen with Nutlin-3 treatment at equivalent concentrations. An increase in the percentage of Annexin V positively stained cells over time reflected the decrease in viability for cell lines expressing wt-p53 (Figure 3; shown as Mean only because of space restriction). A summary of the Annexin V positive data for both wt-p53 cell lines is presented in Table 2.

**Table 2:** Summary of Annexin positive cells in wtp-53 lymphoma cell lines exposed to HDM2-SMIs

**Cell line**	**Time (h)**	**Conc. (μM)**	**Nutlin-3**	**MI-219**	**ANOVA p**^**a**^	**Post-hoc p**^**b**^
WSU-FSCCL	12 h	0	5.3 ± 0.7	5.3 ± 0.7	p = <0.001	--
		2.5	7.0 ± 2.5	13.7 ± 1.3		p = <0.001
		5.0	8.8 ± 2.0	23.8 ± 2.8		p = <0.001
		10.0	10.6 ± 1.7	36.1 ± 2.3		p = <0.001
	24 h	0	6.2 ± 0.7	6.0 ± 0.7	p = <0.0001	--
		2.5	9.6 ± 1.6	27.6 ± 8.2		NS
		5.0	12.3 ± 2.4	43.0 ± 18.6		p = <0.01
		10.0	17.3 ± 4.9	69.7 ± 6.6		p = <0.001
	48 h	0	6.1 ± 1.2	6.1 ± 1.2	p = <0.0001	--
		2.5	10.1 ± 0.7	19.5 ± 1.0		NS
		5.0	12.6 ± 1.9	35.6 ± 7.6		p = <0.001
		10.0	16.5 ± 2.6	74.5 ± 4.9		p = <0.001
KM-H2	12 h	0	7.9 ± 1.2	7.9 ± 1.2	p = 0.026	--
		2.5	11.2 ± 1.6	12.5 ± 1.3		NS
		5.0	11.8 ± 1.5	13.5 ± 0.6		NS
		10.0	13.6 ± 2.4	14.8 ± 2.9		NS
	24 h	0	8.9 ± 1.7	8.9 ± 1.7	p = 0.026	--
		2.5	11.2 ± 3.0	14.2 ± 2.5		NS
		5.0	14.5 ± 3.0	19.9 ± 3.5		NS
		10.0	20.1 ± 3.8	27.9 ± 4.8		p = 0.05
	48 h	0	2.8 ± 2.5	12.8 ± 2.5	NS	
		2.5	15.9 ± 6.5	19.7 ± 9.4		
		5.0	18.9 ± 8.9	23.1 ± 10		
		10.0	21.1 ± 9.7	32.4 ± 9.2		

^a:^ ANOVA for overall significance between all groups. ^b:^ Tukey’s post hoc comparisons between pairs (Nutlin-3 versus MI-219 at equivalent concentrations).

**Figure 4:**
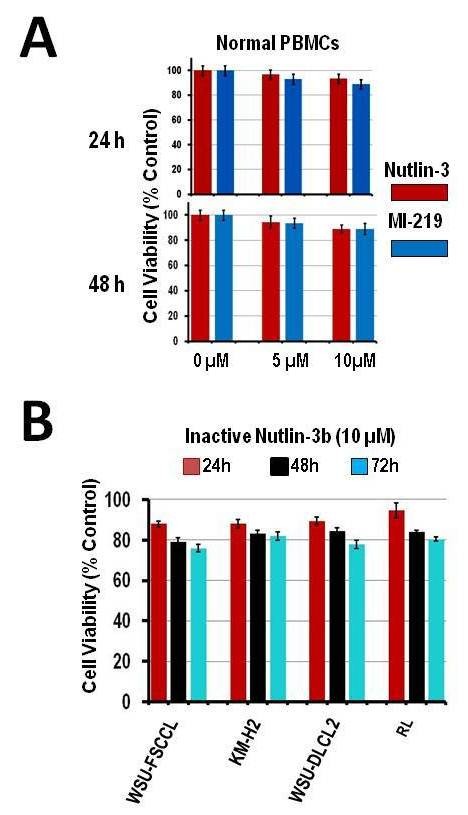
**Effect of HDM2 inhibition in normal B-lymphocytes derived from healthy donors. A**) Isolated B-lymphocytes from normal donors were exposed to HDM2 SMIs for up to 48 h. Neither HDM2 SMI induced a significant decrease in cell viability compared to untreated cells (control). **B**) The inactive Nutlin-3 analog, Nutlin-3b, was ineffective in significantly decreasing cell viability in all 4 cell lines for up to 72 h. Columns and error bars represent Mean ± S.D. of three independent experiments.

Data shown in Figure 4A, indicate that neither HDM2 SMI significantly affected the viability of B-lymphocytes derived from normal donors exposed for up to 48 h. The inactive Nutlin-3 enantiomer, Nutlin-3b, did not show any significant reduction in the cell viability of cell lines, demonstrating the selectivity of each HDM2 SMI (Figure 4B)*.*

### Evaluation of p53 and p53-dependent target proteins

**Figure 5:**
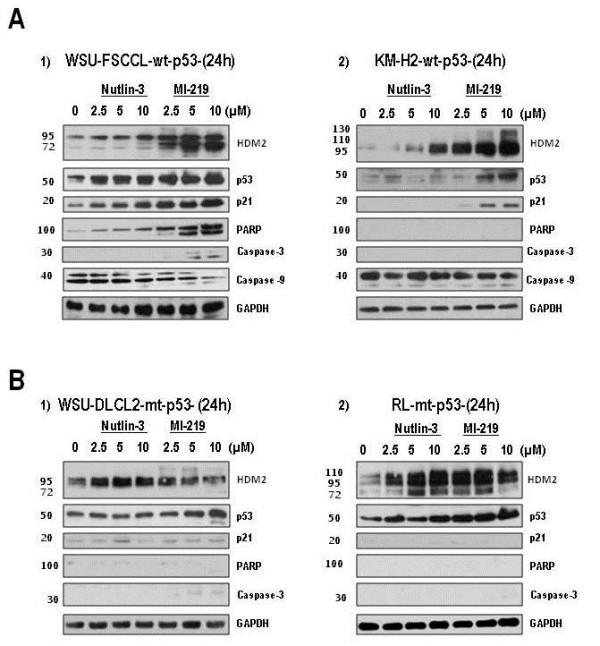
**Upregulation of p53 and p53-target proteins upon HDM2 inhibition.** Cells were exposed to increasing concentrations of HDM2 SMIs for 24 h. Whole cell lysates were subjected to SDS-PAGE and probed for specific proteins. Western blots show changes in p53 and its target proteins after 24 h for the 4 cell lines studied. **A**) The two wt-p53 cell lines, WSU-FSCCL (A1) and KM-H2 (A2) are presented in the top panel. **B**) The two mt-p53 cell lines, WSU-DLCL_2_ (B1) and RL (B2) are presented in the lower panel. Representative blots are shown.

HDM2 inhibitors upregulate expression of p53-dependent target proteins in wt-p53 cell lines after 24 h (Figure 5). Similar to that observed in patient’s samples, this time point best captured the differences between treatments and demonstrated the effects of MI-219 and Nutlin-3 in modifying the expression of p53, p21, cleaved PARP, cleaved Caspase-3 and Caspase-9 in wt-p53 cell lines. Once again, differences in response to these HDM2 SMIs were evident between the two wt-p53 cell lines (Figure 5A1 and A2). Of note, there was little change in p53 or p21 protein levels in mt-p53 cells (WSU-DLCL_2_ and RL) and did not show major evidence of PARP and Caspase-3 cleavage (Figure 5B1 and B2). These results show that HDM2 SMIs cannot effectively restore p53 activity to mt-p53 cell lines.

To understand a possible explanation for the enhanced activity of MI-219 over that of Nutlin-3, we determined the IC_50_s of the HDM2 SMIs using viable cell count as the endpoint in cultures of wt-p53 cell lines (WSU-FSCCL and KM-H2) at equal concentrations for both HDM2 inhibitors. Although the IC_50_s at 48 h were similar for both agents in wt-p53 WSU-FSCCL cells (2.5 μM for MI-219 and 3 μM for Nutlin-3), the IC_50_s were significantly different in wt-p53 KM-H2 cells (3 μM for MI-219 and 8 μM for Nutlin-3).

### HDM2 inhibition enhances the posttranslational stability of p53

**Figure 6:**
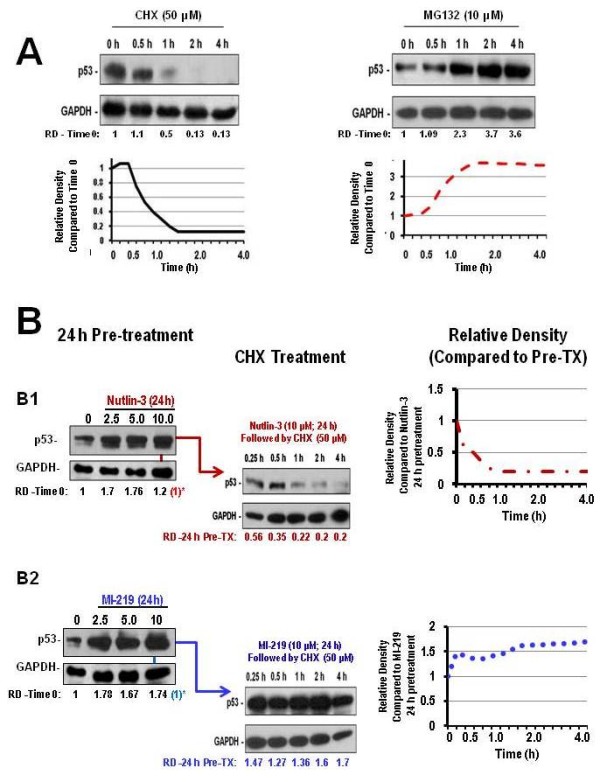
**HDM2 SMIs enhance p53 stability at the posttranslational level. A**) WSU-FSCCL cells were exposed to 50 μM cyclohexamide (CHX) to stop protein translation or 10 μM MG132 to halt proteasome activity over the course of 4 h. **B**) Cells were pre-treated with 10 μM of Nutlin-3 (**B1**) or 10 μM MI-219 (**B2**) for 24 h and then exposed to 50 μM CHX for up to 4 additional hours. Samples were removed at 0.25, 0.5, 1.0, 2.0 and 4 h to evaluate the stability of p53 protein. RD represents relative density to time 0 for CHX and MG132 blots in (**A**) and time at 24 h 10 μM pre-treatment for blots in section (**B**). Changes in relative protein densities are plotted from values obtained (and listed below blots) to show effects of HDM2 SMIs on sustaining p53 protein expression in the presence of added 50 μM CHX.

HDM2 inhibition is hypothesized to increase p53 stability by reducing HDM2-mediated degradation. However, p53 stability could also be the result of enhanced p53 protein translation. To demonstrate that upregulation of p53 protein expression shown in the Western blots were the result of HDM2 inhibition by SMIs, the half-life of p53 was monitored. The inhibition of protein synthesis by treatment with 50 μM CHX alone led to a marked decrease of p53 protein expression over time. Blocking protein translation with CHX decreased the turnover of endogenous p53 (Time 0-2 h; ~t1/2 = 0.68 h) (Figure 6)*.* Furthermore, addition of 10 μM of the proteasome inhibitor MG132 alone ameliorates the degradation of p53, thereby enhancing its stabilization.

Treatment with 10 μM Nutlin-3 or 10 μM MI-219 alone for 24th led to an overall increase in p53 protein expression. Whether p53 stability is related to HDM2 inhibition was evaluated by pre-incubation of 10 μM Nutlin-3 or MI-219 for 24 hours in wt-p53 WSU-FSCCL cells followed by treatment with 50 μM CHX at the indicated time points. Blocking protein synthesis after pre-treatment with HDM2 SMI led to an overall increase in p53 protein expression. Intriguingly, MI-219 treatment was more effective in enhancing p53 stability than Nutlin-3. Pre-treatment with 10 μM Nutlin-3 barely extended the p53 stability in the presence of CHX compared to 10 μM Nutlin-3 alone (Time 0-2 h;~ t1/2 =0.86 h) (Figure 6B1) whereas 10 μM MI-219 greatly enhanced the overall stabilization of p53 protein despite the presence of CHX (Figure 6B2).

### HDM2 inhibition upregulates p53-dependent genes in wt-p53 lymphoma cell lines

**Figure 7:**
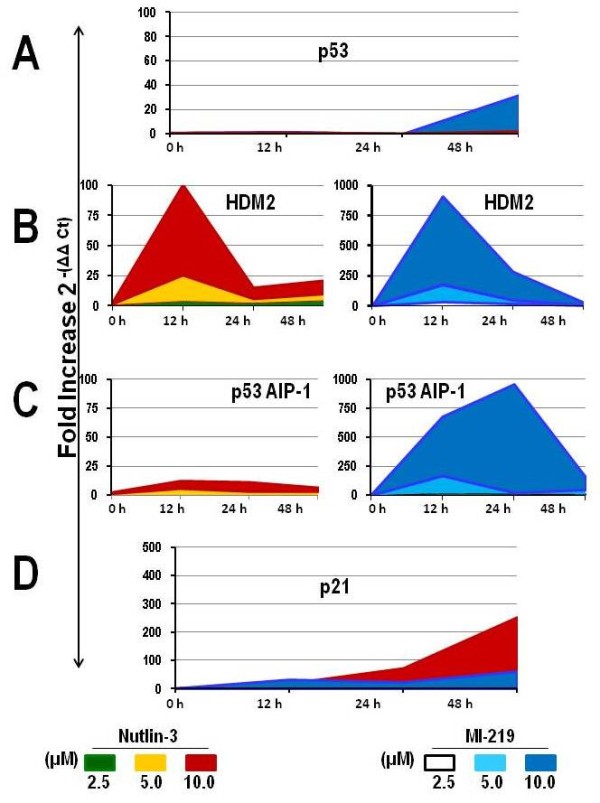
**Effect of HDM2 inhibition on p53-dependent gene expression.** WSU-FSCCL cells were exposed to increasing concentration of Nutlin-3 and MI-219 for 12, 24 and 48 h. Baseline gene expression and after treatment were quantified by qRT-PCR relative to GAPDH using the ΔΔCt method and expressed as fold induction of gene expression relative to that in the untreated control. mRNA expression levels for p53 (**A**), HDM2 (**B**), p53AIP1 (**C**) and p21 (**D**). Coded colors represent different concentrations and are listed for each HDM2 SMI at the base of the page. Error bars plotted represent mean values ± SE performed in triplicate from two independently treated experiments.

To investigate the effects of HDM2 inhibition on p53 transcriptional regulation, we assessed the effect of SMI-mediated reactivity of p53 to enhance target gene expression levels using qRT-PCR. Additionally, we wanted to determine whether the increase in p53 was the result of newly transcribed p53 mRNA or the accumulation of p53 resulting from the HDM2-p53 disruption. Wt-p53 WSU-FSCCL cells exhibited increases in p53-target genes HDM2, p21, p53AIP1 upon HDM2 inhibition compared to control cells albeit with variable kinetics The results are presented in Figure 7. Of particular importance is that there was virtually no upregulation of p53 mRNA transcripts after treatment with HDM2 SMIs at 24 h, suggesting stabilized p53 protein is the result of HDM2 SMIs and not due to enhanced mRNA transcribed into protein. Interestingly, p53 transcripts mRNA increased 29-fold at 48 h in cells exposed to 10 μM MI-219 compared to a 2.5-fold increase in cells exposed to 10 μM Nutlin-3 for the same time period. Overall, MI-219 treatment demonstrated a surprisingly greater induction of p53-target genes compared to Nutlin-3. There was much higher and more sustained induction of HDM2 mRNA by MI-219 compared with Nutlin-3. Effect on HDM2 transcript peaked at 12 hours but was still remarkable at 24 hour in MI-219-treated cells. Both agents induced upregulation of p21 mRNA in WSU-FSCCL cells with overall higher induction by Nutlin-3 compared with MI-219. However, MI-219 effect was more evident at the earlier time points (12 and 24 hours) and at lower concentrations (2.5 and 5 μM) compared with Nutlin-3. The later induced a delayed induction (48 hours) of p21mRNA. p21 mRNA was increased up to 65-fold at 48 h in cells exposed to 10 μM MI-219 but was increased up to 252-fold at the same time period in cells exposed to 10 μM Nutlin-3. MI-219 was more effective than Nutlin-3 in inducing p53AIP1 mRNA, indicating greater induction of p53-dependent apoptosis genes.

### MI-219, but not Nutlin-3, enhances HDM2-medicated autoubiquitination and degradation

**Figure 8:**
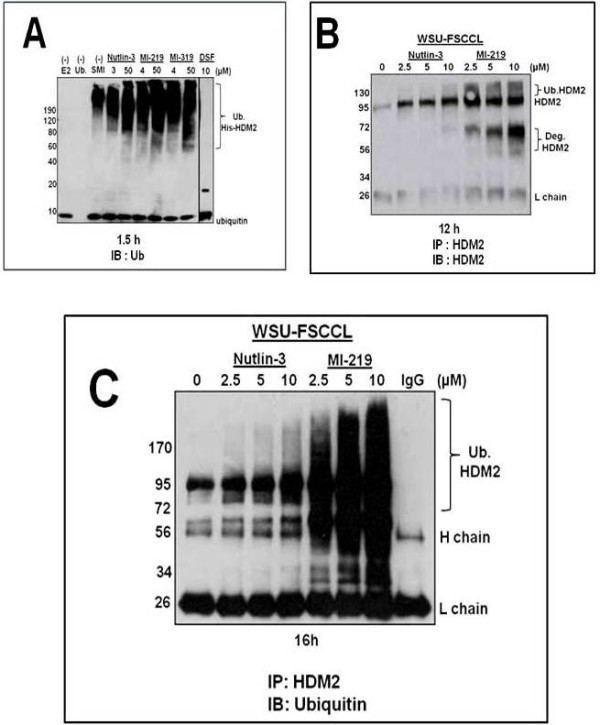
**MI-219, but not Nutlin-3, enhances HDM2-mediated autoubiquitination and degradation. A**) Immunoblot probed for ubiquitin represent autoubiquitination of recombinant His-HDM2 in the presence of Nutlin-3, MI-219 or MI-319 (laboratory grade MI-219) at IC_50_ or 50 μM for 1.5 h in a cell free autoubiquitination assay. Lane 1: no E2 enzyme; Lane 2: no ubiquitin; Lane 3: Control, no SMI. HDM2 SMIs were assessed at IC_50_ or 50 μM concentrations and were as follows; Lane 4–5, Nutlin-3; Lanes 6–7, MI-219; Lanes 8–9, MI-319; Lane 10: Disulfiram (10 μM). **B**) Western blot for HDM2 immunoprecipitated from WSU-FSCCL cells exposed to HDM2 SMIs for 12 h was then probed for with HDM2 to expose full length, ubiquitinated and degraded HDM2 species as described in Methods. **C**) Immunoprecipitated HDM2 from WSU-FSCCL cells exposed to HDM2 SMI for 16 h was immunoblotted with monoclonal anti-ubiquitin antibody to reveal ubiquitin associated-HDM2. Data are representative of 3 independent experiments.

Neither class of agents inhibited the E3 ligase function of HDM2 in a cell-free ubiquitination assay (Figure 8A). Autoubiquitination of recombinant His-HDM2 was not inhibited by the addition of Nutlin-3, MI-219 or MI-319 (laboratory grade MI-219) at IC_50_ or even at much higher (50 μM) concentrations for 1.5 h. Disulfiram (10 μM) completely abrogated autoubiquitination and was included as a negative control.

Identification of HDM2 degraded species from WSU-FSCCL cells was confirmed by immunoprecipitation studies described in Methods. HDM2 from cells exposed to increasing concentrations of Nutlin-3 and MI-219 for 12 h was immunoprecipitated using a1:1 ratio of mouse monoclonal antibodies SMP-14 and D-12 (SMP-14 is known to detect the 60 kDa cleavage product of HDM2) and then immunoblotted (IB) with HDM2 polyclonal antibody (AF1244) to revealed the full length HDM2 in addition to higher molecular weight species (~130 kDa bands [likely autoubiquitinated HDM2]) and ~60 kDa band (the major degraded species of HDM2) (Figure 8B). The intensity of the degraded HDM2 bands were concentration-dependent when obtained from MI-219-treated cells but this was not seen in HDM2 extracted from Nutlin-3-treated cells.

To determine to what extent each of the two HDM2 SMIs induced autoubiquitination of HDM2 in WSU-FSCCL, cells were treated with increasing concentrations of Nutlin-3 and MI-219 for 16 h. Prior to cell harvesting, cells were exposed to MG132 (10 μM) for an additional 15 minutes to preserve the ubiquitinated proteins. HDM2 was immunoprecipitated using antibodies to HDM2 as described above. The blots were then immunoblotted with monoclonal anti-ubiquitin antibody to detect ubiquitinated HDM2. Blots shown in Figure 8C demonstrate that MI-219 induced far greater dose dependent autoubiquitination of HDM2 than Nutlin-3 in this cell line.

These findings suggest that MI-219 but not Nutlin-3 posttranslationally regulates HDM2 protein by inducing autoubiquitination and degradation of itself as a way of compensating for the high levels of HDM2 produced in response to activated p53.

### Comparison of MI-219-induced HDM2 autoubiquitination and degradation in KM-H2 versus WSU-FSCCL cell lines

**Figure 9:**
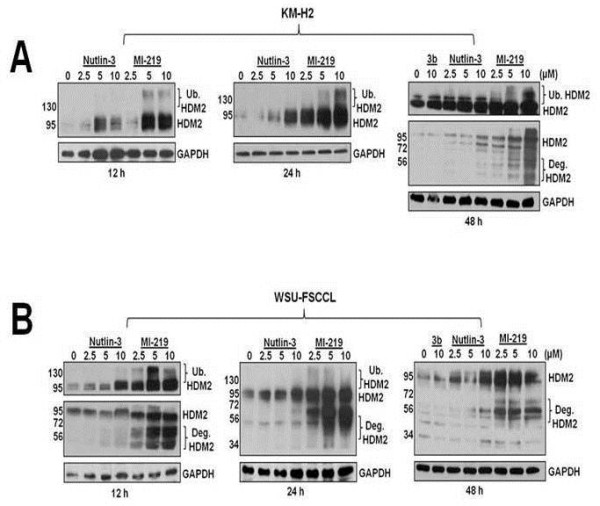
**Activation of HDM2 autoubiquitination and degradation in wt-p53 cell lines exposed to MI-219.** Western blots show that patterns of autoubiquitination and degradation differ between the wt-p53 cell lines. **A**) Data obtained from KM-H2 cells and **B**) WSU-FSCCL cells exposed to HDM2 SMIs for up to 48 h.

We confirmed our autoubiquitination and degradation observations in the KM-H2 cell line, which had previously appeared to be less sensitive to the effects of both HDM2 inhibitors. As can be seen in Figure 9A, MI-219 induced a more robust response inducing HDM2 autoubiquitination and degradation than Nutlin-3 in both cell lines. However, in KM-H2 cells, HDM2 degradation was delayed compared to that observed in the WSU-FSCCL cell line (Figure 9B).

## Discussion

In unstressed cells, p53 is tightly regulated by HDM2 to ensure that its anti-proliferative and anti-apoptotic activity will not harm normal cells. HDM2 and p53 exist in a finely tuned balanced auto regulatory feedback loop by which the two proteins mutually control their cellular levels [27]. In many cancers, this balance is skewed when overexpression of HDM2 leads to constant suppression of p53 posttranslational modifications (such as phosphorylation, acetylation, methylation) which are necessary for the p53 response to stress. Although the majority of lymphoma tumors retain wt-p53 status, mechanisms to compromise p53 signaling, such as HDM2 overexpression, deletions in p14ARF, and viral oncogenes exist to prevent p53 activation in response to stress or initiate programmed cell death [28-30]. Because of the importance of HDM2 in regulating p53 function, attempts are being made to test the effects of its inhibition in cancer. The discovery of small molecular inhibitors of HDM2-p53 interaction is considered a significant development in the treatment of all types of cancers, particularly those in which the activation of wt- p53 is suppressed. These small molecules are designed to physically sit in the p53 binding pocket of HDM2, thereby preventing HDM2-p53 interaction [31]. HDM2 inhibitors R7112 (Nutlin-3 analog) and JNJ-26854165 (a tryptamine derivative) are currently under clinical evaluation in Phase I studies (NCT00623870, NCT00559533, NCT00676910) and the first results from Phase 1 pharmacokinetic and pharmacodynamic study for JNJ-26854165 or Serdemetan in patients with advanced solid tumors has recently been published [32]. These first results indicate that this agent showed clinical efficacy but at elevated doses some toxic effect were of concern. The authors concluded newer derivatives would provide more optimal results.

We had previously investigated MI-319 in preclinical studies using lymphoma cell lines with encouraging results and found that MI-219, MI-319 and Nutlin-3 exhibited similar activity against the cell lines at the concentrations used in this study [26]. Here, we investigated selected effects of MI-219, a clinical grade spiro-oxindole [33] against lymphoma cell lines and patient-derived primary indolent non-Hodgkin’s SLL/CLL lymphoma samples. Using the *cis*-imidazoline inhibitor, Nutlin-3 as a benchmark for comparison, we noted significant differences between the two classes which may have clinical implications. Compared to Nutlin-3, differences could be attributed to enhanced MI-219-induced HDM2 autoubiquitination and degradation which resulted in higher levels of p53 expression and p53 stabilization at equivalent concentrations. Our results indicate that the enhanced MI-219-induced increase in p53 is the result of disruption of the HDM2-p53 complex and released p53 rather than *de novo* transcribed or translated p53. Further in-depth studies with the WSU-FSCCL cell line demonstrated that MI-219 enhanced efficacy was associated with significantly increased p53-induced p53AIP1 mRNA at the expense of p53-induced HDM2 mRNA at 24 h which was not seen with Nutlin-3. Nutlin-3 induce a greater increase in p53-induced p21 mRNA than MI-219 at 48 h. These results provide evidence that MI-219 can induce cell death pathways earlier and more robustly than Nutlin-3 in cell lines and in patient samples. The enhanced effects of MI-219 could be attributed to several factors including higher binding affinity reported for MI-219 to HDM2 (7-fold higher than Nutlin-3) [29], differences in chemical structure or differences in solubility between the two agents. Although in our previous study, we found the binding affinity of MI-219 to be only ~3 times higher than that for Nutlin-3, it is highly unlikely that a 3-fold difference is crucial as both HDM2 inhibitors are over 500 times more potent than the natural p53 peptide [26]. Polar groups added to MI-319 to produce the structure of MI-219, increased its solubility for better dispersal in aqueous media and resulted in increased biological activity [33]. The relationship between aqueous solubility and activity has also been demonstrated in the development of another HDM2 SMI, HLI373, which was found to be more potent than its insoluble parent compound HLI 98 s [34]. Three p53 amino acids (Phe19, Trp23 and Leu26) have been found to be essential for binding between the two proteins and are inserted into a deep pocket on the surface of HDM2. Nutlin-3 mimics the p53 binding pocket at these three amino acids. In contrast, MI-219 mimics 4 key binding residues in p53 (Phe 19, Leu22, Trp23 and Leu26) resulting in more optimal hydrogen binding and hydrophobic interaction with HDM2 and this alone may account for its increased efficacy.

While it is known that p53 is induced in response to a number of stimuli including DNA damage, if p53 is bound to HDM2, that response is suppressed. During DNA damage, in normal cells, HDM2 can be phosphorylated at multiple sites which leads to HDM2 ubiquitination, degradation and p53 stabilization [35,36]. Once again, in cells in which HDM2 is overexpressed, more HDM2 protein can quickly bind to and suppress p53 activation. The focus of our study here was to demonstrate that HDM2 inhibition using HDM2 SMIs would allow free p53 to be activated, if and when necessary, in response to internal as well as external stimuli or stress. Because HDM2 inhibition with these HDM2 SMI does not involve a response to DNA damage, the question remains as to what then initiates the activated p53-induced death pathways in lymphoma cells once p53 suppression is released. Are they the same factors encountered by normal cells and is the response the same for cancer and non-cancerous cells are question for future studies. However, p53 is not the only partner for HDM2. There are more than 30 known proteins that form interactions with HDM2 and include proteins such as NF-κB, p21,pRb, TGFβ-1 as well as others [37]. Their function and significance are not fully understood, although they may have therapeutic implications and have been shown to play a role in cellular responses such as transcriptional regulation, apoptosis and the cell cycle [15,38].

In addition to disrupting the HDM2-p53 interaction, there is evidence that HDM2 SMIs may induce posttranslational modification of p53. Poyurovsky et al. recently showed that Nutlin-3 induced the modification of the C-terminus of p53 (including acetylation) which may explain its lower efficiency in dissociating p53-HDM2 in vitro [39]. In other studies, using solid tumors, MI-219 was found to target the class II histone deacetylase SIRT 1 (which deacetylates p53 allowing HDM2 to target p53 to ubiquitination) and the Bax-Ku70 interaction [40]. Suppression of Ku70, in theory, would allow pro-apoptotic Bax localization to mitochondria and p53-mediated apoptosis.

Nutlin-3 was one of the first HDM2-SMIs to show significant efficacy in a number of models, thus, there are more published reports on this agent than on MI-219. Bixby et al. confirmed that p53 status was the most important predictor of response to HDM2 SMI in isolated CLL cells studied ex vivo [41]. Results of other studies indicate CLL cells exposed to Nutlin-3 ex vivo in combination with cytotoxic chemotherapeutic agents have demonstrated synergy; while activity of Nutlin-3 as single agent, was modest [42]. Ex vivo studies using isolated multiple myeloma cells indicate that Nutlin-3 was as effective as melphalan without the genotoxic effects [43]. In treatment resistant acute myelogenous leukemia cell, Nutlin-induced apoptosis was mediated by transcriptional activation of pro-apoptotic Bcl-2 family proteins and transcriptional-independent mitochondrial permeabilization via mitochondrial p53 translocation [44]. In Hodgkin lymphoma samples, Nutlin-3 was able to mediate p53 stabilization, cell cycle arrest and initiate the apoptotic death pathway, including Caspase-3 and PARP cleavage.

Differences in response to Nutlin-3 and MI-219 between the WSU-FSCCL and KM-H2 cell lines were seen in our study and may reflect biological differences between Hodgkin’s and non-Hodgkin’s lymphomas which they represent, including different time-frame of response to these two HDM2 SMIs.

This study is one of the first, if not the first, to compare the effects of Nutlin-3 and MI-219 in SLL/CLL samples ex vivo. These SLL/CLL and MZL samples represent slowly dividing tumor cells which have lost the ability to undergo programmed cell death or apoptosis and this leads to bone marrow replacement by lymphoma which impedes its function. Agents which affect mitotic or cell cycle pathways are generally not useful as a therapeutic regimen. Instead, optimal anti-cancer agents are sought to quickly initiate cell death processes for these lymphomas while sparing normal cells. The population studied here exhibits varied genetic characteristics and varied responses to standard treatment and thus, offered an excellent opportunity to test the effects of HDM2 SMIs for treatment of this disease. The results show that the response to MI-219-induced HDM2 autoubiquitination and degradation and associated increase in p53 varied among the studied samples and reflected the genetic diversity which has thwarted the development of a single cure for this group. In other studies, failure of CLL to respond upregulation of p53 have been attributed to polymorphism in the p21 gene [45], transactive defective spliced variants of the p53 gene [46], and altered p53-induced effectors [47].

For extended experimental research studies, we utilized cell lines derived from patients with aggressive forms of NHL. Since these cells do proliferate, effects of anti-cancer agent modifying cell cycle associated proteins (such as p21) can be demonstrated. Further studies in wt-p53 WSU-FSCCL NHL cell line confirmed that MI-219 induced enhance HDM2 autoubiquitination and degradation compared to that in Nutlin-3-treated cells. As this was a consistent finding observed in both wt-p53 cell lines and in patient samples, we demonstrated that none of the HDM2 SMIs (MI-219, MI-319 or Nutlin-3) inhibited recombinant HDM2 ubiquitination and degradation by themselves in a cell-free ubiquitination assay. This indicates that the E3 ligase function of HDM2 is not affected by the inhibitors. The exact mechanism for enhanced MI-219 induced HDM2 autoubiquitination will require further detailed investigation. Possible mechanisms include binding of MI-219, but not Nutlin-3, to HDM2 results in its interaction with one of the many proteins that are known to bind to, and modulate activity of HDM2. Some of these HDM2-interacting proteins have been shown to facilitate or induce HDM2 autoubiquitination and include L11, 14-3-3σ, SCL-BP1, FKBP25, ZNF668, TR3, and RASSF1A [48-54]. In addition, anticancer agents such as Parthenolide accelerate HDM2 autoubiquitination through mechanisms that required ATM and Berberine, which downregulates HDM2 at the posttranslational level through modulation of death domain-associated protein (DAXX) have recently been shown to induce apoptosis [55,56].

## Conclusions

In conclusion, our study reveals for the first time unexpected differences between MI-219 and the prototypical Nutlin-3 in lymphoma cell lines and patient samples. We propose a novel mechanism for MI-219 anti-lymphoma activity that alters the functional activity of HDM2 through enhanced autoubiquitination and degradation. Additionally, this mechanism appears to correspond to biological outcome. Our results provide evidence that different classes of HDM2 SMIs elicit molecular events that extend beyond HDM2-p53 dissociation which may be of biological and potentially therapeutic importance given the oncogenic nature of HDM2. Further investigation of such interaction is pivotal to full realization of the therapeutic potential of these agents in cancer therapy.

## Methods

### Human lymphoma cell lines and patient-derived B-lymphoma cells

WSU-FSCCL and WSU-DLCL_2_ cell lines were established in our laboratory as previously described [57,58]. WSU-FSCCL (wt-p53) is a human B-cell follicular small cleaved cell line; WSU-DLCL_2_ (mt-p53) is a human diffuse large B-cell line. The human Hodgkin lymphoma derived cell line KM-H2 (wt-p53) was obtained from DSMZ (Germany). The human diffuse lymphoma cell line RL (mt-p53) was obtained from the American Type Culture Collection (Manassas, VA). Cells from normal, anonymous healthy donors were isolated from discarded apheresis cones obtained from the Red Cross and were kindly provided by Dr. Martin Bluth, Associate Director of Detroit Medical Center Transfusion Services. Peripheral blood was collected from lymphoma patients in leukemic phase following informed consent in the Lymphoma Clinic at St. John Hospital Van Elslander Cancer Center for clinical examination in accordance with institutional review board (IRB) approval. Ethical consideration for this research study to use the discarded blood after clinical analyses was approved following expedited review by the IRB at Wayne State University School of Medicine.

Patient-derived and normal donor peripheral blood mononuclear cells (PBMCs) were isolated by LymphoPrep density gradient centrifugation (ProGen Biotechnik GmbH, Germany). Monocytes were depleted from the LymphoPrep gradient fraction by allowing cells to adhere to a sterile plastic surface for approximately 1–2 hours at 37°C. Depletion of T-lymphocytes was carried out using 100 μl of Dynabeads pan CD2 (Dynal, Life Technologies, Grand Island, NY). Cells were incubated with prewashed beads for 30 min at 4°C while rotating. T-cells were depleted using the DynaMag as a magnetic retrieval device according to the manufacturer’s protocol. The unbound, negatively selected, highly purified B-cell populations were recovered by aspiration and used for functional assays. FACS analysis confirmed non-B-cell depletion and verified that the recovered cell population contained >90% B-lymphocytes. All cells were maintained in suspension in RPMI 1640 medium supplemented with 10% fetal bovine serum (Denville Scientific, Denville, NJ) and 1% Penicillin/Streptomycin (Invitrogen, Carlsbad, CA) at 37°C in a 5% CO_2_ humidified incubator.

### Reagents and drug treatments

MI-219 (Ascenta Therapeutics, Malvern, PA) was synthesized using methods previously published [59,60]. Disulfiram (a RING-finger ubiquitin E3 ligase inhibitor), Nutlin-3 (Sigma Aldrich, St. Louis, MO), its 150-times less active enantiomer (+)-Nutlin-3b (Cayman Chemical, Ann Arbor, MI), and MI-219 were dissolved in 100% DMSO as 10 mM stock solution. The proteasomal inhibitor, MG132 (Cayman Chemical) and cycloheximide (Sigma) were dissolved in 100% DMSO as 100 mM stock solution. Reagents were further diluted in sterile water immediately prior to adding to cultures to obtain the desired final concentration.

### Identification of p53 status

p53 cDNA sequencing was used to identify the p53 status of the patient samples. Genomic DNA was extracted from approximately 1 × 10^6^ purified B-lymphocytes recovered from each patient sample using a ZR-*Duet*™ DNA/RNA MiniPrep kit (Zymogen Biotech). Samples were quantitated by UV absorption at 260 nm and 200 ng was used in each PCR amplification reaction. Two primer sets were used to amplify p53 coding exons 5–9 as previously described [26,61]. These exons have been identified as the DNA-binding region which contains approximately 90% of all known p53 mutations [62]. Amplified cDNA PCR products were analyzed by agarose gel electrophoresis and purified using Wizard SV Gel/PCR Cleanup kit (Promega, Madison, WI). Two primers, one from each primer set, and 200 ng of the PCR products were sent to GeneWiz, Inc. (South Plainfield, NJ) for DNA sequencing. cDNA sequences were analyzed for p53 mutations by comparing the obtained sequences to BLAST wt-p53 coding sequence: NCBI Reference Sequence: NM_000546.4.

Characteristics of the patient’s lymphoma were made available through the Lymphoma Clinic at (St. John’s Hospital) and FISH analyses of chromosomal re-arrangements were provided by Dr. Steve Buck at Children’s Hospital of Michigan Flow Cytometry Core.

### Cell viability assays

Cell lines and patient derived cells were seeded at a density of 2 x 10^5^/ml and allowed to adapt overnight before being exposed to varying concentrations of Nutlin-3 or MI-219 the following day. Control cells were treated with equal volume of DMSO for a final concentration of 0.1%. 3-(4, 5-dimethylthiazol-2-yl)-2, 5-diphenyl-tetrazolium bromide (MTT) reagent (Sigma) was added 24, 48 or 72 hours later to halt reactions and monitor cell viability in cell lines. Purple formazan crystals were solubilized in DMSO and absorbance was read in a plate reader at 540 nM.3-(4, 5-dimethylthiazol-2-yl)-2, 5-diphenyl-tetrazolium bromide (MTT) reagent (Sigma) was added 24, 48 or 72 hours later to halt reactions and monitor cell viability in cell lines. Purple formazan crystals were solubilized in DMSO and absorbance was read in a plate reader at 540 nM. Cell viability was also determined at for patient samples using Trypan Blue (0.4%; Sigma) Exclusion. The IC_50_ was assessed as 50% inhibitory concentration compared to vehicle-treated (control). Data are represented as Mean ± SE. of at least three independent experiments performed in duplicate for each cell line.

Biostatistical analyses assessing percent survival of patient tumor samples were replicated for a minimum of 2 and a maximum of 6 times for each patient at each drug, dose, and time point. Cell survival for the cumulative patient cohort ranged from 0% to 100%. The quantile-quantile plot for patient samples indicated that the observed data had a longer right tail than would be expected if the data were normally distributed. Nonetheless, a mixed effects analysis of variance was used because the violation was not outrageously extreme and because no better alternative could be found given the experimental design. In the mixed effects model drug, concentration and time were defined as fixed effects; patient and replication were defined as random effects. Holm’s procedure was used to adjust for multiple comparisons.

### cDNA preparation and quantitative real-time PCR to detect the induction of p53-target genes

Total RNA was extracted from treated and control lymphoma cells using the PureLink™ RNA Mini Kit (Invitrogen). Total RNA was quantified by NanoDrop and 1 μg of each sample was reverse-transcribed using the SuperScript® VILO™ cDNA synthesis kit according to the manufacurer’s instructions (Invitrogen). The resulting cDNA preparations were then cleaned of excess enzyme with Wizard SV Gel/PCR Cleanup kit (Promega). Real time PCR amplifications were conducted in a 10 μl reaction volume using the Roche LightCycler® 480 SYBR Green I Master (Roche, Pleasanton, CA) according to manufacturer’s protocol. 100 ng cDNA samples were used for each reaction and mixed with Quanti Tect Primers (Qiagen) to amplify p53, HDM2, p21, p53AIP1 using GAPDH as the internal standard. Qiagen priority patened primer sequences verified and standardized for specific gene products were those supplied with Qiagen Tect sets; (http://www.qiagen.com). Reactions were carried out in a 384-well microtiter plate using the LightCycler® 480 System (Roche). Two independent drug treatments per sample were performed in triplicate and each reaction was repeated at least once to ensure accuracy. The PCR cycle number at threshold (CT) was used for the comparison. Baseline gene expression and gene expression post treatment were quantified by qRT-PCR relative to GAPDH using the ΔΔCt method and expressed as fold induction of gene expression relative to that in the untreated control [63]. Values represent mean ± SE of two independent experiments performed in triplicate. Statistical analyses were performed by two-way ANOVA using GraphPad Prism v. 4.0.

### Western blots

Treated cells were collected by centrifugation, washed twice with sterile PBS, and solubilized in M-PER lysis buffer (ThermoScientific, Rockford, IL) consisting of a cocktail of protease and phosphatase inhibitors (ThermoScientific). The concentration of total cell lysate was quantified by BCA protein method (Pierce; Thermo Scientific). Cellular lysates (50 μg of protein) were fractionated onto 14% or 4-20% Tris-Glycine SDS-PAGE gels, transferred onto PVDF membrane, and probed with primary antibodies to p53 (DO-1; Santa Cruz Biotechnology, Inc, Santa Cruz, CA), HDM2 (AF1244; R and D Systems, Minneapolis, MN); p21 (2947), cleaved PARP (5625), cleaved caspase-3 (9664) and ubiquitin (3936) from Cell Signaling Technology, Danvers, MA, with GAPDH (Trevigen, Gaithersburg, MD) as the internal control. Secondary antibodies were anti-mouse or anti-rabbit conjugated to HRP (Jackson ImmunoResearch). Proteins were visualized using chemiluminescence substrate reagents.

For statistical analyses of data for primary lymphoma cells, relative density was selected as the endpoint. It was defined as the ratio of the absolute density for a given protein treated with a given drug, at a given concentration, for a specific time, for a specific patient to the absolute density of GAPDH under the same conditions. Fixed effects linear models were used with drugs, Nutlin-3 and MI-219 concentrations, proteins, and time as fixed effects and patient as the random effect. Evaluation of the shape of the frequency distribution of relative density indicated that a natural log transformation was required to meet the assumptions of the statistical tests. Holm’s procedure was used to adjust for multiple comparisons.

### Cell-free HDM2 autoubiquitination assay

Cell free autoubiquitination assays were performed using 200 ng of recombinant His-HDM2 in the presence or absence of E2-conjugatng enzyme, UbcH5b, recombinant human E1 (Boston Biochem, Cambridge, MA), in the presence or absence of HDM2 SMIs. Final concentration of drug was IC_50_ or 50 μM for Nutlin-3, MI-219, and MI-319 diluted in 30 μL reaction volume. Disulfiram (10 μM), an ubiquitin E3 ligase inhibitor was used as a control. Samples were incubated for 1.5 hours at 30°C. The reaction was stopped with the addition of 10 μl of 3X SDS loading dye, boiled for 5 minutes, separated on a 4-20% Tris-Glycine gradient gel (Invitrogen), and then immunoblotted with anti-ubiquitin antibody.

### Immunoprecipitation

WSU-FSCCL or KM-H2 cells were treated with Nutlin-3 or MI-219 for specified period of time, harvested and lysed as described above. Total cellular lysate (1 mg) was incubated with 5 μg primary antibody overnight (a 1: 1 ratio of HDM2 antibodies SMP-14 and D-12, Santa Cruz) rotating at 4°C (SMP-14 is known to detect the 60 kDa degraded product of HDM2). The following day, Protein G agarose beads (Millipore) were added to each sample and incubated at 4°C for an additional 4 hours. Agarose bead-linked antibody: antigen complexes were collected by centrifugation, washed 3 times with 1× PBS prior to the addition of 3× SDS-PAGE loading dye, denatured at 95°C for 5 min, and centrifuged briefly. The solubilized immunoprecipitates were separated on a 4-20% Tris-Glycine gradient gel and immunoblot (IB) with HDM2 polyclonal antibody (AF1244). Ubiquitin antibody was used for co-immunoprecipitation.

### Data analysis and statistical significance

Statistical analyses were performed by two-way ANOVA unpaired two-tailed GraphPad Prism v. 4.0 or unpaired two-tailed *t* test using Microsoft Excel. ImageJ densitometry software (Version 1.45, US National Institutes of Health) was used for quantification of Western blot bands. Selected bands were quantified based on their relative integrated intensities, calculated as the product of the selected pixel area and the mean gray value for those pixels normalized to internal control (GAPDH). Fold increase or decrease was calculated by standardizing each treatment as a ratio to the control. Additional data analysis and statistical methods are described in different sections of the Materials and Methods above. Statistical significance was set at p < 0.05 for all data comparisons.

## Abbreviations

CHX: Cyclohexamide; CLL: Chronic lymphocytic leukemia; DSF: Disulfiram; HDM2: Human Double Minute 2; IB: Immunoblotting; IP: Immunoprecipitation; Mt-p53: Mutant p53; MTT: (3-(4,5-Dimethylthiazol-2-yl)-2,5-diphenyltetrazolium bromide; NHL: Non-hodgkins lymphoma; p53AIP1: p53 apoptosis-inducing protein 1; PBMC: Peripheral blood mononuclear cells; SMI: Small-molecule inhibitor; Wt-p53: Wild-type p53.

## Competing interests

The authors have no conflict of interest to declare and none has any commercial interest in any of the agents used in this study.

## Authors’ contributions

AMS designed and carried out all experiments, correlated and analyzed the resulting data, organized and prepared the manuscript for publication. AMB provided the reagents and supervised the cell-free ubiquitination assays that AMS performed in her lab as well as interpretation of data. AS provided technical assistance to AMS in carrying out experiments. JA provided biostatistical analyses of the primary cell data. RMM provided direction, guidance, interpretation and critiquing of data. AMA provided suggestions, additional funding, lymphoma patient samples and encouragement for the overall project. All authors read and approved the final manuscript.

## Authors’ information

Grant support: Ruth L. Kirschstein National Research Service Award T32-CA009531 (A. M. Sosin).
